# Useful outcome measures in INPH patients evaluation

**DOI:** 10.3389/fneur.2023.1201932

**Published:** 2023-08-07

**Authors:** Laura Mori, Federica Collino, Annalisa Marzi, Lucia Pellegrino, Marta Ponzano, Davide Del Chiaro, Sara Maestrini, Stefano Caneva, Matteo Pardini, Pietro Fiaschi, Gianluigi Zona, Carlo Trompetto, Ligurian INPH Study, Giulia Biasotti

**Affiliations:** ^1^Department of Neuroscience, Rehabilitation, Ophthalmology, Genetics, Maternal and Child Health, University of Genoa, Genoa, Italy; ^2^IRCCS Ospedale Policlinico San Martino, Genoa, Italy; ^3^Department of Health Sciences (DISSAL), University of Genoa, Genoa, Italy

**Keywords:** hydrocephalus, normal pressure, walking, balance, gait analysis, outcome

## Abstract

**Introduction:**

Idiopathic normal pressure hydrocephalus (INPH) is a neurological disorder that is potentially reversible and clinically characterized by a specific triad of symptoms, including gait disturbance, cognitive disorders, and urinary incontinence. In INPH assessment, the most commonly used test is the Timed Up and Go test (TUG), but a more comprehensive assessment would be necessary. The first aim of the present study is to verify the sensitivity of a protocol with both clinical and instrumental outcome measures for gait and balance in recognizing INPH patients. The second aim is to verify the most important spatio-temporal parameters in INPH assessment and their possible correlations with clinical outcome measures.

**Methods:**

Between January 2019 and June 2022, we evaluated 70 INPH subjects. We assessed balance performances with the Berg Balance Scale (BBS), Short Physical Performance Battery (SPPB), and TUG, both single (ST) and dual task (DT). We also performed an instrumental gait assessment with the GAITRite electronic walkway system, asking the patients to walk on the carpet for one minute at normal speed, fast speed, and while performing a dual task. We compared the results with those of 20 age-matched healthy subjects (HS).

**Results:**

INPH patients obtained statistically significant lower scores at the BBS, SPPB, and TUG DT but not at the TUG ST, likely because the DT involves cognitive factors altered in these subjects. Concerning instrumental gait evaluation, we found significant differences between HS and INPH patients in almost all spatio-temporal parameters except cadence, which is considered a relevant factor in INPH guidelines. We also found significant correlations between balance outcome measures and gait parameters.

**Discussion:**

Our results confirm the usefulness of BBS and suggest improving the assessment with SPPB. Although the TUG ST is the most commonly used test in the literature to evaluate INPH performances, it does not identify INPH; the TUG DT, instead, might be more useful. The GAITRite system is recognized as a quick and reliable tool to assess walking abilities and spatio-temporal parameters in INPH patients, and the most useful parameters are stride length, stride width, speed, and the percentage of double support. Both clinical and instrumental evaluation may be useful in recognizing subjects at risk for falls.

## 1. Introduction

Idiopathic normal pressure hydrocephalus (INPH) is a neurological disorder characterized by ventricular dilation visible by brain imaging and normal cerebrospinal fluid (CSF) pressure during lumbar puncture. Clinically, it is characterized by a specific triad of symptoms including gait disturbance, cognitive disorders, and urinary incontinence (1, 2). INPH manifests during adult life as an insidiously progressive, chronic disorder that lacks an identifiable antecedent cause (3).

Due to the limited knowledge of pathophysiological mechanisms, non-specific symptoms, and the high prevalence of comorbidities, INPH is largely underdiagnosed (4). A group of Norway researchers highlighted that ~5% of dementia diagnoses were caused by INPH (3).

In this disease, the accumulation of CSF is the cause of the symptoms (5), and its withdrawal may lead to an improvement in all the symptoms (6, 7). A large multicenter study reports improvements in the gait domain in 77% of the patients, 63% in the domains of neuropsychology, 56% in balance, and 66% in urinary continence, after CSF shunt surgery (8). Therefore, INPH diagnosis is very important since it is considered a neurological disorder potentially reversible in which a gait disorder and dementia can improve to a complete remission of symptoms especially if detected and treated early (9–13).

In the INPH guidelines, a diagnosis is probable whether the patients present a suggestive clinical history with an insidious onset, a minimum duration of at least 3 months, no other disorders causing the symptoms, ventricular enlargement evidenced at brain imaging study (CT or MRI), CSF opening pressure in the range of 5–18 mm Hg (or 70–245 mm H_2_O) as determined by a lumbar puncture and the presence of gait or balance disturbance (3, 14), and at least one other area of impairment in cognition, urinary symptoms, or both.

With respect to gait/balance, at least two of the following should be present:

1. Decreased step height.
2. Decreased step length.
3. Decreased cadence.
4. Increased trunk sway during walking.
5. Widened standing base.
6. Toes turned outward on walking.
7. Retropulsion (spontaneous or provoked).
8. *En bloc* turning (turning requiring three or more steps for 180 degrees).
9. Impaired walking balance, as evidenced by two or more corrections out of eight steps on tandem gait testing.

Patients with INPH manifest some or all the classic clinical symptoms in a variable way, but often the first disorder is represented by gait disorders (15). In most studies, walking impairment is evaluated just by clinical observation or based on the patient's impression of improvement, but both are subjective measures, therefore prone to bias (10, 16–18). In the literature, the most used test is the timed up and go test (TUG) (19, 20), which mainly investigates dynamic balance, but, as mentioned, INPH symptomatology is very complex, and the TUG may not be sufficient to capture improvements in the other domains, both motor and cognitive (21). The availability of objective outcome measures is very important in every disease. INPH is even more relevant to assess the improvements after CSF subtraction to predict the possible results after shunt surgery. For a comprehensive evaluation, it is necessary to find clinical and instrumental outcome measures that may help physicians in performing an objective and correct evaluation of INPH subjects and evaluating the possible improvements after CSF subtraction. For this reason, in our movement analysis laboratory, we evaluated the gait and balance of INPH patients with a protocol of both clinical and instrumental outcome measures. The first aim of the present study is to confirm the sensitivity of clinical and instrumental outcome measures for gait and balance in recognizing INPH patients. The second aim is to verify the most important spatio-temporal parameters in INPH assessment and their possible correlations with clinical outcome measures.

## 2. Materials and methods

This retrospective study was based on data obtained from patients attending the multidisciplinary outpatient clinic for the diagnosis and treatment of INPH at the IRCCS Ospedale Policlinico San Martino of Genoa, Italy. The study was conducted according to the guidelines of the Declaration of Helsinki.

The inclusion criteria included those as follows: confirmed clinical and neuroradiological diagnosis of INPH (3); adults; ability to walk without support for at least 25 m; Short Physical Performance Battery (SPPB) scoring between 2 and 10; and ability to sign informed consent.

The exclusion criteria included those as follows: other forms of neurological disorders; vestibular affections; psychiatric, cardiovascular, and lung disorders or severe arthropathic changes in the lower limbs; and inability to perform the TUG.

Between January 2019 and June 2022, we evaluated 70 subjects affected by INPH, who met all the inclusion criteria. All patients underwent an MRI with stroke volume evaluation, and after recording a detailed medical history, a complete neurosurgical, neurological, and physical examination was performed. A single examiner was responsible for the assessment of all subjects. All subjects underwent an evaluation by means of clinical scales and instrumental gait evaluation.

We also dispose data from a control group of 20 healthy age-matched subjects (HS).

### 2.1. Clinical outcome measures

Balance performances have been evaluated with the Berg Balance Scale (BBS), SPPB, and TUG for both single and dual tasks.

Particularly, the TUG is a test widely used to assess the possible improvements after CSF withdrawal (tap test) in INPH patients. It involves rising from a seated position, walking 3 m, turning around, walking back, and sitting back down. This test is a simple and quick measure of functional mobility and has excellent reliability among healthy older adults and populations with different medical diagnoses (22). In elderly persons, the mean (95% confidence interval) TUG time for individuals between 60 and 90 years of age is lower than 10 s (23, 24). The cognitive task in TUG DT, in our protocol, a serial-3 subtraction task, involves sustained attention, information processing speed, and working memory abilities (21, 25). Previous studies document that the time difference between dual- and single-task TUG is a valid marker of frailty and falls (21, 26).

The BBS, a 14-item objective test, is a sensitive scale to detect subtle balance impairment and fall risk that has been validated in people affected by neurological disorders (27–30) and has also been used in the assessment of disability in patients affected by INPH (19, 31, 32). The total score ranges from 0 to 56, but a score below 45 is indicative of imbalance and a great risk of falls (33, 34).

SPPB is a widely used instrument to quantify balance disorders and gait impairment in elderly people, in many different neurological diseases (28, 29, 35–38), and in INPH patients (39). It is a composite measure assessing walking speed, standing balance, and sit-to-stand performance, which has been shown to have a high level of validity, reliability, and responsiveness in measuring physical function (40). The total score ranges from 0 to 12, and a score below 10 is associated with a risk of falls (41, 42).

### 2.2. Instrumental evaluation

We performed an instrumental gait assessment by means of the GAITRite electronic walkway system, which is a 7-m-long electronic portable walkway able to measure the temporal and spatial gait parameters. The electronic walkway is non-invasive and does not require any attachments to the subject under investigation.

As the subject ambulates across the walkway, the pressure exerted by the feet onto the walkway activates the sensors. Our patients were asked to walk on the carpet for 1 min at normal speed (normal walk, NW), at fast speed, but not running (fast walk, FW), and at normal speed enunciating aloud all possible words beginning with a chosen letter (dual task, DT). Patients always had a researcher walking alongside them as a safeguard.

PKMAS is software in conjunction with the GAITRite system, providing information about spatial and temporal parameters of the objects in contact with the walkway surface.

As seen in the literature regarding the most used parameters (43–46), we focused our attention on the following: stride length, stride width, stride time, velocity, and cadence. We also checked for the different percentages of the gait cycle time: stance, swing, single support, and total double support.

Stride length is the distance from the heel of one foot to the following heel of the same foot (cm). Stride width is the distance between a line connecting the two ipsilateral foot heel contacts (the stride) and the contralateral foot heel contact between those events and is measured perpendicular to the stride (cm). Stride time and gait cycle time are the period of time from the first contact of one foot to the following first contact of the same foot (s). Stance time is the period of time when the foot is in contact with the ground (s). Stance percentage is the stance time presented as a percentage of the gait cycle time. Swing time is the period of time when the foot is not in contact with the ground (s). Swing percentage is swing time presented as a percentage of the gait cycle time. Single support time is the period of time when only the current foot is in contact with the ground (s). Single support percentage is single support time presented as a percentage of gait cycle time. Total double support time is the sum of all periods of time when both feet are in contact with the ground during the stance phase (s). Total double support percentage is total double support time presented as a percentage of the gait cycle time. Velocity is obtained after dividing the sum of all stride lengths, by the sum of all stride time (cm/s). Cadence is the number of footfalls minus one, divided by the ambulation time (steps/min).

### 2.3. Statistical analysis

The sample size for the control group was calculated based on a two-sample means test, fixing power to 0.90 and alpha to 0.05. The sample size was assessed for the evaluations under study (stride length, double sup, velocity, and stride width), fixing the number of cases and mean (SD) of the evaluations based on the observed values among cases and setting the effect sizes based on preliminary results on a subgroup of available controls. The sample of controls was designed to be comparable to cases in terms of age (age categories).

We verified that cases and controls were comparable for age by performing the two-sample *t*-test, subsequently, we compared the two groups in terms of stride length, double sup, velocity, stride width, BBS, SPPB, and TUG using *t*-test, and we calculated the Cohen's *d* to describe the standardized mean difference of an effect (47). We also calculated Pearson's correlation coefficients between balance outcome measures and gait parameters among cases (48).

A two-sided α of <0.05 was considered to be statistically significant. All statistical analyses were performed using Stata version 16.0 (Stata Corporation, College Station, TX, USA).

## 3. Results

We analyzed the data of 70 INPH patients attending our movement analysis laboratory.

The mean age was 75.5 ± 5.8 years (men 60%, women 40%).

We also confronted the scores with a group of age-matched healthy subjects (75.1 ± 5.1 years), free of neurological comorbidities, who attended our outpatient service for osteoporosis management. The exclusion criteria were severe osteoporosis, neurological disorders, fractures, or previous surgery at lower limbs or who were not able to walk without support for at least 25 m; we also included caregivers and carers who met the same criteria.

Table 1 provides a summary of clinical outcome measures.

**Table 1:** Clinical and demographical characteristics of INPH patients and HS.

	**Cases (*N* = 70)**	**Controls (*N* = 20)**	***p*-value**	**|Cohen's *d*|**	**Area under ROC curve**
Age, mean (SD)	75.51 (5.79)	75.15 (5.13)	0.8000	0.064	
Male, *N* (%)	42 (60%)	5 (25%)	**0.006**	0.757	
**Scale**					
BBS, mean (SD) (*N* = 88)	40.84 (12.38)	53.65 (2.41)	**<0.001**	1.167	0.902
SPPB, mean (SD)	4.70 (3.10)	8.85 (2.64)	**<0.001**	1.381	0.837
TUG, mean (SD) (*N* = 88)	23.44 (14.94)	16.28 (24.99)	0.1147	0.405	0.815
TUGDT, mean (SD) (*N* = 88)	33.52 (26.41)	17.61 (6.61)	**0.0093**	0.677	0.779

BBS, Berg Balance Scale; SPPB, short physical performance battery; TUG, timed up and go test; DT, Dual Task; SD, standard deviation; INPH, idiopathic normal pressure hydrocephalus; HS, Healthy Subjects. The bold values indicate the significant *p* values.

We found a significant difference between INPH patients and HS in all scales (*p* < 0.05) except TUG ST.

Table 2 provides a summary of the instrumental gait assessment.

**Table 2:** Instrumental gait assessment.

**NW (*N* = 85)**	**Cases (*N* = 65)**	**Controls (*N* = 20)**	***p*-value**	**|Cohen's *d*|**	**Area under ROC curve**
Stride length, mean (SD)	76.97 (24.72)	108.76 (17.41)	**<0.001**	1.367	0.845
Double support, mean (SD)	43.11 (9.45)	34.77 (4.15)	**0.0003**	0.977	0.778
Velocity, mean (SD)	60.88 (22.82)	87.05 (17.78)	**<0.001**	1.202	0.815
Stride width, mean (SD)	12.56 (3.53)	7.46 (2.93)	**<0.001**	1.501	0.867
Step length, mean (SD)	8.43 (12.40)	54.45 (8.92)	**<0.001**	1.369	0.842
Step time, mean (SD)	0.64 (0.12)	0.57 (0.06)	**0.0250**	0.584	0.681
Stride time, mean (SD)	1.28 (0.25)	1.15 (0.12)	**0.0253**	0.582	0.673
Stance, mean (SD)	71.46 (4.84)	67.15 (2.01)	**0.0002**	0.999	0.780
Swing, mean (SD)	28.57 (4.79)	32.85 (2.01)	**0.0002**	0.992	0.780
Single support, mean (SD)	28.49 (4.66)	32.60 (1.97)	**0.0003**	0.978	0.771
Cadence, mean (SD)	94.57 (16.92)	97.11 (15.31)	0.5507	0.153	0.545
**FW (*****N*** = **82)**	**Cases (*****N*** = **62)**	**Controls (*****N*** = **20)**	***p*** **-value**	**|Cohen's** ***d|***	**Area under ROC curve**
Stride length, mean (SD)	88.96 (28.22)	120.82 (20.86)	**<0.001**	1.195	0.821
Double support, mean (SD)	39.01 (8.72)	31.15 (4.12)	**0.0002**	0.999	0.773
Velocity, mean (SD)	81.16 (30.90)	111.41 (29.63)	**0.0002**	0.988	0.754
Stride width, mean (SD)	11.77 (3.53)	7.33 (2.89)	**<0.001**	1.309	0.853
Step length, mean (SD)	44.39 (14.11)	60.37 (10.55)	**<0.001**	1.197	0.822
Step time, mean (SD)	0.56 (0.08)	0.51 (0.05)	**0.0162**	0.632	0.671
Stride time, mean (SD)	1.12 (0.17)	1.02 (0.11)	**0.0116**	0.664	0.686
Stance, mean (SD)	69.47 (4.34)	65.31 (1.91)	**0.0001**	1.067	0.793
Swing, mean (SD)	30.55 (4.34)	34.79 (1.91)	**0.0001**	1.087	0.798
Single support, mean (SD)	30.54 (4.38)	34.36 (2.14)	**0.0003**	0.965	0.770
Cadence, mean (SD)	107.91 (17.06)	111.02 (17.33)	0.4826	0.181	0.549
**DT (*****N*** = **84)**	**Cases (*****N*** = **64)**	**Controls (*****N*** = **20)**	***p*** **-value**	**|Cohen's** ***d|***	**Area under ROC curve**
Stride length, mean (SD)	71.96 (25.41)	101.81 (13.41)	**<0.001**	1.288	0.852
Double support, mean (SD)	46.11 (11.20)	35.79 (4.04)	**0.0001**	1.031	0.798
Velocity, mean (SD)	55.67 (25.10)	71.76 (17.66)	**0.0093**	0.682	0.711
Stride width, mean (SD)	13.55 (4.58)	8.31 (3.57)	**<0.001**	1.199	0.831
Step length, mean (SD)	35.96 (12.67)	51.05 (6.82)	**<0.001**	1.304	0.852
Step time, mean (SD)	0.66 (0.16)	0.64 (0.10)	0.7063	0.097	0.500
Stride time, mean (SD)	1.32 (0.31)	1.31 (0.27)	0.9332	0.022	0.500
Stance, mean (SD)	73.08 (5.55)	67.49 (2.10)	**<0.001**	1.124	0.820
Swing, mean (SD)	27.02 (5.46)	32.51 (2.10)	**<0.001**	1.122	0.820
Single support, mean (SD)	27.01 (5.49)	32.11 (1.98)	**0.0001**	1.038	0.796
Cadence, mean (SD)	91.30 (19.82)	85.49 (17.19)	0.2416	0.302	0.607

NW, normal walk; FW, fast walk; DT, dual task; SD, standard deviation. The bold values indicate the significant *p* values.

A total of five patients were excluded from the analysis because they were not able to walk on the carpet for a minute. The other three patients were not able to modify the speed at the FW, and one patient was excluded from the analysis because he refused to perform the DT.

At the NW and FW, we found significant differences between INPH patients and HS in all spatio-temporal parameters except for cadence.

At DT, we found significant differences in all parameters except for step, stride time, and cadence.

Large effect size based on Cohen's *d* (*d* ≥ 0.8) was observed for stride length (NW: *d* = 1.367, FW: *d* = 1.195, DT: *d* = 1.288), double support (NW: *d* = 0.977; FW: *d* = 0.999; DT: *d* = 1.031), velocity (NW: *d* = 1.202; FW: *d* = 0.988), stride width (NW: *d* = 1.501; FW: *d* = 1.309; DT: *d* = 1.199), step length (NW: *d* = 1.369, FW: *d* = 1.197; DT: *d* = 1.304), stance (NW: *d* = 0.999, FW: *d* = 1.067; DT: *d* = 1.124), swing (NW: *d* = 0.992; FW: *d* = 1.087; DT: *d* = 1.122), and single support (NW: *d* = 0.978; FW: *d* = 0.965; DT: *d* = 1.038).

**Figure 1:**
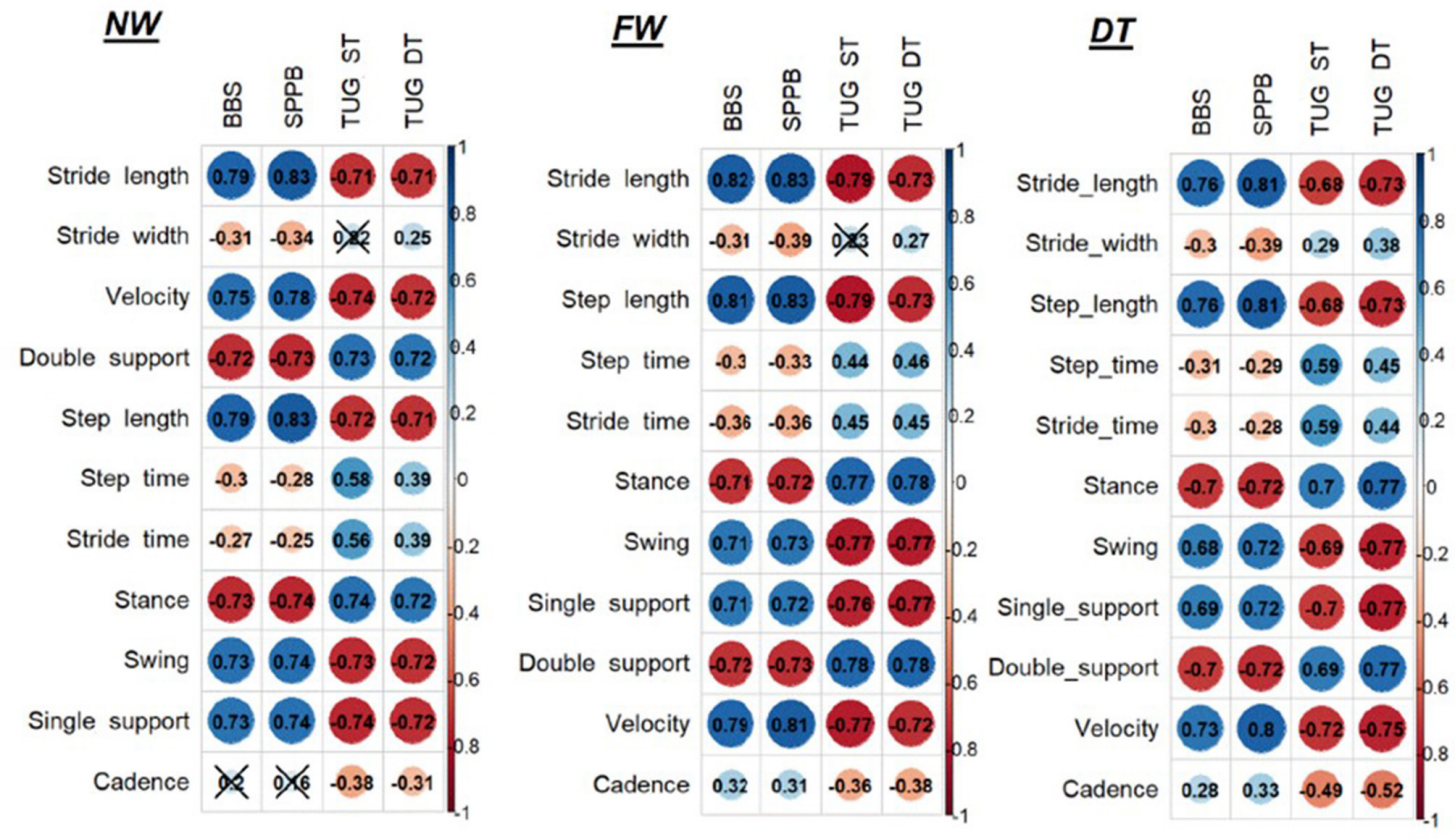
Correlations between clinical outcome measures and instrumental spatio-temporal parameters. NW, Normal walk; FW, Fast walk; DT, Dual task; BBS, Berg Balance Scale; SPPB, Short Physical Performance Battery; TUG, Timed up and go test; ST, Single task.

Regarding the correlations between clinical outcome measures and instrumental spatio-temporal parameters, see Figure 1.

At the NW and FW, we found that stride length and velocity have a high significant correlation with BBS and SPPB and a high negative correlation with TUG both ST and DT (*p* < 0.001); stride width has only a low negative correlation with BBS and SPPB (*p* < 0.05). Concerning the percentages of gait phases, we found a high significant correlation between all the parameters: the percentage of double support and stance showed a high negative correlation with BBS and SPPB (*p* < 0.001), a high positive correlation with TUG ST and DT (*p* < 0.001), while the percentage of swing and single support present high positive correlation with BBS and SPPB (*p* < 0.001) and high negative correlation with TUG both ST and DT (*p* < 0.001).

At DT, we found that stride length has a high positive correlation with BBS and SPPB (*p* < 0.001), high negative correlation with TUG DT (*p* < 0.001), and moderate to high negative correlation with TUG ST (*p* < 0.001); velocity has a high positive correlation with BBS and SPPB and a negative high correlation with TUG both ST and DT (*p* < 0.001); stride width has only low negative correlation with BBS and SPPB (*p* < 0.05). Concerning the percentages of gait phases, we found a high negative correlation between double support percentage with BBS and SPPB, high positive correlation with TUG DT, and moderate to high positive correlation with TUG ST (*p* < 0.001); stance presents high negative correlation with BBS and SPPB (*p* < 0.001), high positive correlation with TUG ST and DT (*p* < 0.001); swing has moderate to high positive correlation with BBS, high correlation with SPPB, moderate to high negative correlation with TUG ST, and high negative correlation with TUG DT (*p* < 0.001); single support has a moderate to high positive correlation with BBS, high correlation with SPPB, and high negative correlation with TUG ST and DT (*p* < 0.001).

The cognitive evaluation performed on a subgroup of 44 subject is presented in Table 3. The MMSE score of 25 shows a moderate cognitive impairment, while the scores at the TMT both A and B and at SDMT show an impairment in the executive function, sustained attention and processing speed. Unfortunately, we cannot draw conclusions because we do not dispose of the data of the same control group.

**Table 3:** Cognitive evaluation performed on a subgroup of INPH subjects.

	**Mean (SD)**
MMSE (*N* = 44)	25 (3.48)
TMT-A (*N* = 43)	90.84 (54.72)
TMT-B (*N* = 34)	212.85 (123.77)
TMT-B-A (*N* = 34)	141.79 (98.98)
SDMT (*N* = 38)	15.76 (10.76)

MMSE, mini mental state examination; TMT, trail making test; SDMT, symbol digit modalities test; INPH, idiopathic normal pressure hydrocephalus.

The MMSE score of 25 shows a moderate cognitive impairment. Some patients failed to perform some test (TMT A and B or SDMT) because they did not understand the task or could not do it. Nevertheless, TMT A and B and SDMT scores show that our patients present with difficulties in executive function, sustained attention, and processing speed.

## 4. Discussion

All our patients reported walking impairment, imbalance, and cognitive disorders such as memory loss and difficulty in attention or concentration. At the clinical evaluation, they presented a mean score lower than 45 at the BBS, a score lower than 10 at the SPPB, and they took more than 20 s at the TUG ST, which are related to imbalance and risk of falls (20, 34, 41, 42, 49, 50). Lower scores on the SPPB have been shown to be predictive of an increased risk of falling, loss of independence in activities of daily living, decreased mobility, and disability (39–41). This may lead to important considerations to achieve in their treatment, such as the adoption of a cane or a walker to improve walking stability and safety.

It is hence not surprising to find significant differences between HS and INPH persons in the balance outcome measures since these subjects often complain imbalance and frequent falls.

BBS and SPPB are confirmed as useful outcome measures for a rapid and reliable clinical assessment of balance (27, 51–53) and also in INPH assessment.

The TUG is a test widely used in INPH and other various neurological pathologies (19, 20, 43, 54, 55). Particularly, in INPH, it has been used as a marker of the efficacy of CSF subtraction. The international guideline for the prevention of falls in frail elderly individuals recommends TUG as a screening tool for increased risk of falls. Previous studies (20, 55, 56) have suggested that elderly individuals scoring 20 s or more on the TUG have a significantly higher risk for falls and recommend this test as a reliable and simple quantitative examination tool for evaluating improvement in gait disturbance and physical performance after the tap test or shunt surgery in INPH. However, our results suggest that the assessment of dynamic balance through the TUG ST test does not show significant differences between INPH subjects and the control group. This may depend on the fact that the control group was constituted of persons of an age at risk of falling due to their frailty because of sarcopenia, osteoporosis, and multiple comorbidities (57–63). Nevertheless, previous studies reported that TUG ST performances could be influenced by age, sex, body height (21, 23, 64), lower limb strength, walking speed (65), and cognitive variables (66, 67), while dual-task tests might have an added value for fall prediction than single-task tests (26, 68–71). Executive function, sustained attention, information processing speed, and functional mobility may contribute to DT performances (21, 25), as well as attention shifting and working memory (72, 73). The TUG DT, in fact, manages to capture significant differences with respect to the healthy sample because the cognitive factor, predominant in the INPH, influences the evaluation (21, 72). Furthermore, previous research showed that the time difference in performances on TUG DT and ST is a valid predictor of frailty and falls (26, 66).

Our results suggest the usefulness of BBS and SPPB in INPH assessment, while the TUG ST is not the better test to perform. To include a double task is important to recognize these patients, given the presence of both motor and cognitive difficulties in INPH.

Concerning instrumental gait evaluation, we found significant differences between healthy subjects and INPH patients in almost all spatio-temporal parameters. Cadence is considered a relevant parameter in INPH assessment, so much so that it is included in the guidelines for the probable diagnosis of INPH (3). However, our results suggest that cadence is not able to detect differences with the control group of HS, and it is hence not suitable to consider it in these subjects' assessments. Instead, reduced stride length and wide standing base, which are also included in the INPH diagnosis guidelines, in our sample are significantly different with respect to the control group, confirming that the most relevant spatio-temporal parameters to consider in INPH subjects are stride length, stride width, and speed; furthermore, we found significant differences between the two groups in the percentage of swing, single and double support phases. All these parameters are related to imbalance. Studies investigating gait impairment and imbalance suggest that double support percentage is considered a stabilizing factor during normal gait in the elderly (15, 44). It is known that faller patients walk with shorter steps, longer stance phase, and a shorter swing phase than the non-fallers (44), and shorter steps result in reduced gait speed. This indicates that the faller patients may use a more conservative and cautious gait strategy to maintain dynamic balance by reducing gait velocity and taking shorter steps to prevent falls; the faller patients may increase the double support period to stabilize their inefficient gait control (44).

The fact that all gait parameters in INPH subjects have strong correlations with all balance scales, both static and dynamic, confirms what is already known, i.e., that patients with imbalance should reduce walking speed and stride length, increasing the double support to increase stability, thus compromising the fluidity of the gait. The stride width, particularly, has been generally considered as a balance-related gait parameter (74), and INPH subjects present a wider base with respect to the healthy group even though there is no correlation with balance outcome measures. This pattern has been also interpreted as a cautious strategy to maintain balance and prevent falls (44). These correlations are similar in all tasks (NW, FW, and DT), suggesting the utility of the GAITRite system in evaluating these patients and the added value of a comprehensive assessment using different gait paradigms.

Concluding, in INPH motor assessment it is important to evaluate both balance and gait performances and BBS and SPPB are confirmed as fast and simple tests accurately evaluating the functional impairment of these patients. Although the TUG ST is the most used test in literature to evaluate INPH performances, it does not show significant differences between INPH subjects and the control group, and this may be explained because it could be influenced by age, sex, lower limb strength, and walking speed while dual-task tests might have an added value since executive function, sustained attention, information processing speed, and functional mobility are involved.

The GAITRite system is a quick and reliable tool to assess walking abilities and spatio-temporal parameters also in INPH patients. Even though decreased cadence is considered a contributing parameter to the diagnosis of INPH, we found no significant differences compared to the HS group. We suggest that the most useful parameters to consider are stride length, stride width, speed, and the percentage of double support. Both clinical and instrumental evaluation may be useful in recognizing subjects at risk for falls. Our results are encouraging to verify the usefulness of this combined assessment before and after CSF withdrawal to identify subjects who can benefit the most, and, therefore, candidates for the ventricular shunt intervention, thus helping the clinicians in the operative decision.

## Data availability statement

The raw data supporting the conclusions of this article will be made available by the authors, without undue reservation.

## Ethics statement

Ethical review and approval was not required for the study on human participants in accordance with the local legislation and institutional requirements. The patients/participants provided their written informed consent to participate in this study.

## Author contributions

LM was responsible for research design, data analysis, and wrote the manuscript. MPo was responsible for the statistical analysis. CS, GB, LV, and OC performed clinical or instrumental evaluations. FC, AM, LP, SM, and DDC performed all clinical and instrumental evaluations and contributed to the data interpretation. SC performed the cognitive evaluations. EP contributed to the gait analysis. MPa and CT revised the manuscript. PF, PM, and GZ being expert neurosurgeons, recruited the subjects, and revised the manuscript. All authors contributed to the article and approved the submitted version.

## Ligurian INPH study group

Giulia Biasotti^1, 2^, Oscar Crisafulli^1^, Salvatore Fede^2^, Paolo Merciadri^2^, Elisa Pelosin^1^, Elena Saretti^1, 2^, Cristina Schenone^1, 2^ and Lucilla Vestito^1, 2^.

^1^Department of Neuroscience, Rehabilitation, Ophthalmology, Genetics, Maternal and Child Health, University of Genoa, Italy.

^2^IRCCS Ospedale Policlinico San Martino, Genoa, Italy.
